# Skin sensitizers differentially regulate signaling pathways in MUTZ-3 cells in relation to their individual potency

**DOI:** 10.1186/2050-6511-15-5

**Published:** 2014-02-11

**Authors:** Ann-Sofie Albrekt, Henrik Johansson, Anna Börje, Carl Borrebaeck, Malin Lindstedt

**Affiliations:** 1 Department of Immunotechnology, Lund University, Medicon Village building 406, 223 81 Lund, Sweden; 2 Department of Chemistry and Molecular Biology, Dermatochemistry and Skin Allergy, University of Gothenburg, SE-412 96 Gothenburg, Sweden

**Keywords:** Pathways in skin sensitization, Skin sensitizer, *in vitro* assay, Signaling pathways, Metabolic pathways

## Abstract

**Background:**

Due to the recent European legislations posing a ban of animal tests for safety assessment within the cosmetic industry, development of *in vitro* alternatives for assessment of skin sensitization is highly prioritized. To date, proposed *in vitro* assays are mainly based on single biomarkers, which so far have not been able to classify and stratify chemicals into subgroups, related to risk or potency.

**Methods:**

Recently, we presented the Genomic Allergen Rapid Detection (GARD) assay for assessment of chemical sensitizers. In this paper, we show how the genome wide readout of GARD can be expanded and used to identify differentially regulated pathways relating to individual chemical sensitizers. In this study, we investigated the mechanisms of action of a range of skin sensitizers through pathway identification, pathway classification and transcription factor analysis and related this to the reactive mechanisms and potency of the sensitizing agents.

**Results:**

By transcriptional profiling of chemically stimulated MUTZ-3 cells, 33 canonical pathways intimately involved in sensitization to chemical substances were identified. The results showed that metabolic processes, cell cycling and oxidative stress responses are the key events activated during skin sensitization, and that these functions are engaged differently depending on the reactivity mechanisms of the sensitizing agent. Furthermore, the results indicate that the chemical reactivity groups seem to gradually engage more pathways and more molecules in each pathway with increasing sensitizing potency of the chemical used for stimulation. Also, a switch in gene regulation from up to down regulation, with increasing potency, was seen both in genes involved in metabolic functions and cell cycling. These observed pathway patterns were clearly reflected in the regulatory elements identified to drive these processes, where 33 regulatory elements have been proposed for further analysis.

**Conclusions:**

This study demonstrates that functional analysis of biomarkers identified from our genomics study of human MUTZ-3 cells can be used to assess sensitizing potency of chemicals *in vitro,* by the identification of key cellular events, such as metabolic and cell cycling pathways.

## Background

Allergic contact dermatitis (ACD) is an inflammatory skin disease characterized by eczema and recurrent episodes of itching [1]. Hundreds of chemicals are known to cause skin sensitization, making ACD the most common immunotoxic condition in humans. ACD affects a significant proportion of the population. In a recent populations-based investigation, it was demonstrated that the prevalence of ACD is increasing, from 7.2% to 12.9% in the study population [2]. According to the REACH (Registration, Evaluation, and Authorization of Chemicals) regulation [3], chemicals within the EU that are produced or imported (>1 ton/year) must be tested for human hazardous effects. Historically, new chemicals have been tested on animals. For cosmetic ingredients, animal testing is now prohibited [4], even if validated alternative assays are unavailable.

The Local Lymph Node Assay (LLNA) [5], which is currently the gold standard in hazard classification for sensitization, has been shown to have an accuracy of prediction of 72% relative to human data, the same as for the Guinea Pig Maximization Test (GPMT) [6]. Taken together, the ethical aspects of animal testing, the need for monitoring of hazardous effects and the discrepancy between animal and human immune mechanisms makes the need for *in vitro* tests urgent.

Recently, we presented an *in vitro* test, Genomic Allergen Rapid Detection (GARD), based on MUTZ-3 cells [7,8], that was able to accurately classify compounds as sensitizers or non-sensitizers, using a genomic biomarker signature [9,10]. Furthermore, we reported that sensitizers induced a heterogeneous transcriptional response in MUTZ-3 cells, indicating that different sensitizers affect different pathways. The OECD guidelines on adverse outcome pathways [11] attempts to summarize the biochemical pathways that are associated with skin sensitization, listing the activation of inflammation and cellular stress related pathways as critical events. However, transcriptional data from MUTZ-3 cells indicate that the regulation of biochemical pathways might be more diverse than previously described. This led us to further investigate the signaling pathways in relationship to the chemical reactivity properties of each individual skin sensitizer. Consequently, the aim of the current study was to identify adverse outcome pathways associated with different sensitizers, stratified into various chemical reactivity groups, in order to enhance the understanding of the skin sensitization processes in relation to sensitizer potency.

## Methods

### Transcriptional data set

**Figure 1:**
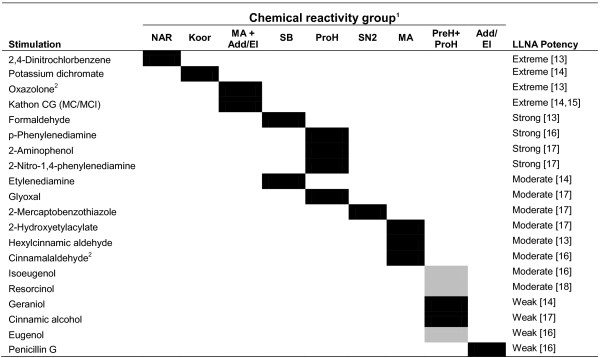
**Stratification of chemical sensitizers into reactive mechanism groups.** 1) Black: classification generally acknowledged. Grey: possibly ambiguous classification. 2) Indicated stimulations did not pass QC tests and were removed from analysis. Chemical reactivity abbreviations: Add/El: Addition/elimination to carboxylic acid derivatives; Koor: Coordination bonds; MA: Michael addition or 1,4-Addition to a/b unsaturated carbonyl; NAR: nucleophilic aromatic substitution, PreH: Prehapten; Chemical activation (outside the skin); ProH: Prohapten; Enzymatic activation; SB: Schiff’s Base reaction; SN2: bimolecular nucleophilic substitution.

A panel of 40 chemical compounds, consisting of 20 sensitizers and 20 non-sensitizers were used for cell stimulations of the human myeloid leukemia-derived cell line MUTZ-3 (DSMZ, Braunschweig, Germany), as detailed earlier in [10]. RNA was isolated, processed to cDNA and hybridized to microarrays (Affymetrix®, Santa Clara, CA) [12]. In short, RNA from un-stimulated and chemically stimulated MUTZ-3 cells, from triplicate experiments, were extracted and analyzed. The preparation of labeled sense DNA was performed, according to Affymetrix® GeneChip® Whole Transcript (WT) Sense Target Labeling Assay (100 ng Total RNA Labeling Protocol), using the recommended kits and controls (Affymetrix®). Hybridization, washing and scanning of the Human Gene 1.0 ST Arrays were performed according to the manufacturer’s protocol (Affymetrix®). 18 of the 20 sensitizing chemicals [13-18] passed QC test after array analysis and were subsequently used for functional analysis (Figure 1). The complete data set consisting of 137 samples has been deposited to ArrayExpress with the accession number [E-MTAB-670].

### Microarray data analysis and statistical methods

The microarray data set was normalized and quality checked, using the RMA algorithm in the Affymetrix® Expression Console software (Affymetrix®). Sensitizers were assigned to at least one of the following chemical reactivity mechanisms; addition/elimination to carboxylic acid derivatives (Add/El), coordination bonds (Koor), Michael addition or 1,4-addition to a/b unsaturated carbonyl (MA), nucleophilic aromatic substitution (NAR), Schiff’s base reaction (SB), biomolecular nucleophilic substitution (SN2), Prehapten (PreH) and Prohapten (ProH). In the present cohort of sensitizers, 9 different groups of chemical reactivity combinations were identified, as shown in Figure 1. The 9 reactivity groups were statistically tested against a pool of non-sensitizing stimulations and vehicle controls (83 samples in total), using ANOVA. The top 1000 genes from each statistical test were identified, and the significance level at which these top genes were found was recorded. All filtering and statistical tests were performed in Qlucore Omics Explorer 2.3 (QOE; Qlucore AB, Lund, Sweden).

### Functional analysis of chemical reactivity groups in IPA®

Each of the 9 gene lists identified with statistical testing was used as input in Ingenuity Pathways Analysis ver. 12710793, as of June 2012 (IPA**®**; Ingenuity Systems Inc., Redwood City, CA), using fold change and p-values as decision values for significance and enrichment calculations. For each data set the significant canonical pathways, significant transcription regulators and cellular functions identified by IPA**®** Core Analysis of Human data using default settings were exported and analyzed further in QOE. Hierarchical Cluster Analysis (HCA) was performed using non-normalized data with maximum linkage.

### Pathway annotation and grouping for assessment of biological functions

All pathways were named according to the terminology used by IPA**®**. In addition to pathway names, we also grouped pathways into functional groups, according to the grouping used by IPA**®** (apoptosis signaling, cell cycle regulation etc.) with all pathways being classified as either signaling or metabolic pathways or both. When a pathway was ambiguously assigned we chose the first listing, according to alphabetical order. Findings were confirmed, using MetaCore™ (Thomson Reuters, NY).

### Availability of supporting data

The microarray data set used was deposited at ArrayExpress with the accession number [E-MTAB-670].

## Results and discussion

### MUTZ-3 transcriptional profiling and stratification of sensitizers into chemical reactivity groups

The transcriptome of MUTZ-3 cells stimulated with skin sensitizers and non-sensitizers was analyzed, using DNA microarray technology, with triplicate samples of 40 individual stimulations [10]. Due to insufficient array quality, 2 out of 20 sensitizers were removed from further analysis, leaving 18 triplicates of sensitizers and 20 triplicates of non-sensitizers for analysis in this study. The relationship between the sensitizers’ potency, according to the Local Lymph Node Assay, and the designation of these into different chemical reactivity groups are summarized in Figure 1.

**Figure 2:**
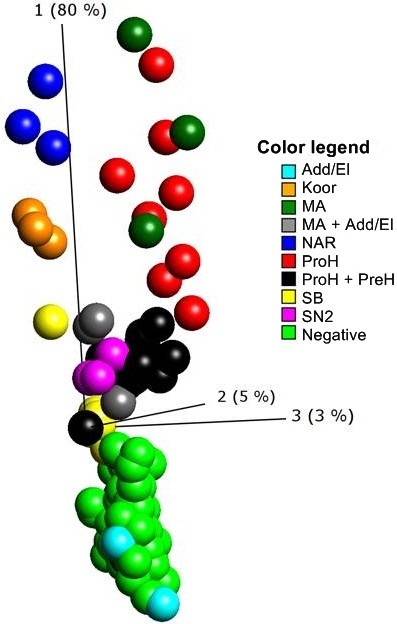
**Principal component analysis (PCA) plot of sensitizers and non-sensitizers.** The PCA plot shows the 26 most significant genes, based on p-value from an ANOVA comparing sensitizers with non-sensitizers. Triplicate samples of 18 sensitizers and 20 non-sensitizers are shown. The coloring shows the different chemical reactivity groups included. The sensitizers are centered in the top of the plot and the non-sensitizers in the bottom of the plot. Add/El: Addition/elimination to carboxylic acid derivatives; Koor: Coordination bonds; MA: Michael addition or 1,4-Addition to a/b unsaturated carbonyl; NAR: nucleophilic aromatic substitution, PreH: Prehapten; Chemical activation (outside the skin); ProH: Prohapten; Enzymatic activation; SB: Schiff’s Base reaction; SN2: bimolecular nucleophilic substitution.

A Principal Component Analysis (PCA) analysis of the complete expression data set visualized that the studied chemical reactivities were indeed quite distinct, as shown in Figure 2. Though there is a certain overlap between the groups there are also clear differences, which is a strong indicator that the 9 chemical groups may trigger different biological activities, while the effects of non-sensitizing samples are homogenous. The PCA plot in Figure 2 was based on the 26 most significant genes in the data set from an ANOVA, comparing sensitizers with non-sensitizers. However, the observed pattern is clearly visible also in the complete data set, where no statistical test has guided the PCA. To further ensure that this is a biological finding, randomization and permutation of data was performed, comparing the original data set to randomized and permuted data sets. Statistical significance was only found in the original data set, which shows that the observed patterns are not seen due to random effects in multidimensional data.

The apparent differences in transcriptional profiles among different reactivity groups led us to further explore the differential engagement of signaling and metabolic pathways and the upstream regulators initiating such pathways. To this end, each chemical reactivity group was compared to the set of non-sensitizers in an ANOVA, yielding 9 lists of differentially regulated genes. These 9 gene lists were subsequently imported into IPA**®** for further analysis.

**Figure 3:**
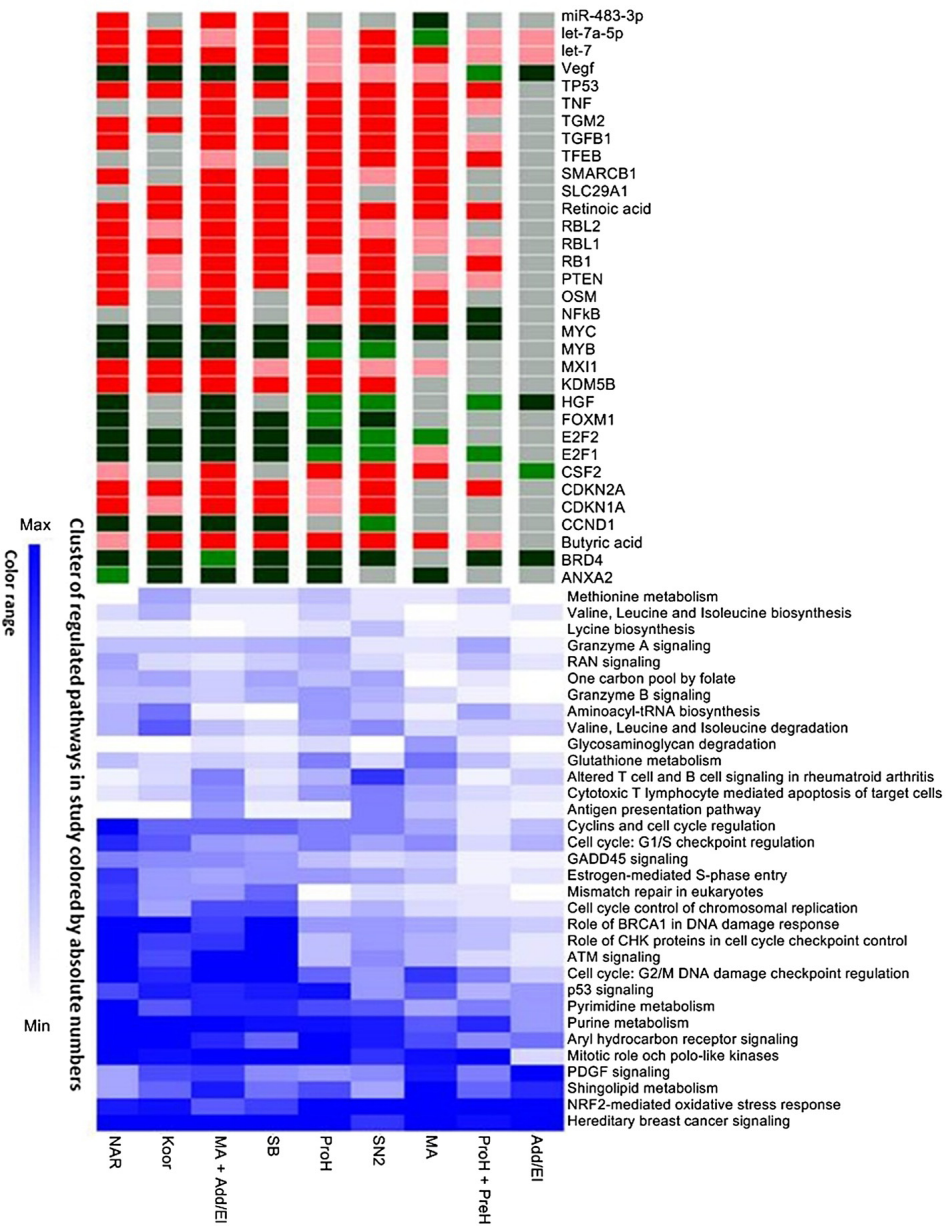
**Hierarchical cluster analysis of pathway and regulatory elements.** This hierarchical cluster analysis shows the regulatory elements predicted to have a general activity in the data set, i.e. in at least 4 of the 9 groups. Regulatory elements are colored according to the activation state. Red color indicates activation, while green color indicates inhibition. Dark and pale red/green colors indicate high and low significance of change, respectively. 33 pathways were found to be significantly regulated by the chemicals. These were clustered and colored from white to blue according to the involvement grade of the pathway, as estimated by the number of regulated molecules. The chemical groups were ordered according to their Local Lymph Node Assay potency with the most potent to the left and least potent to the right in the picture. The complete data set is given in Additional file 1.

**Figure 4:**
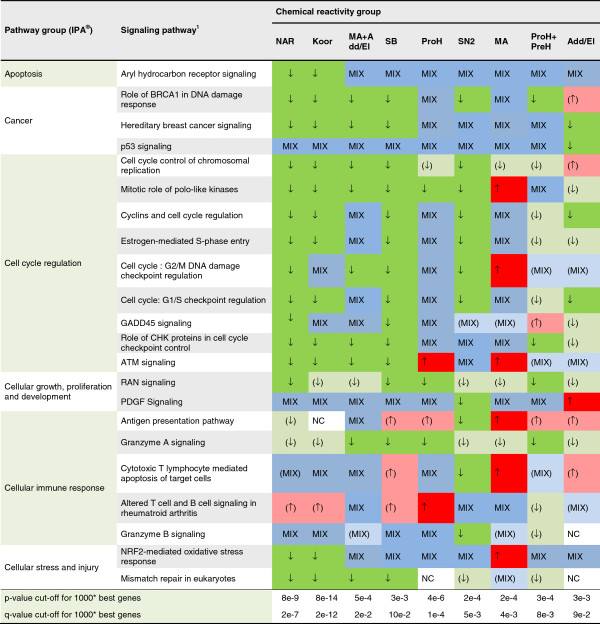
**Signaling pathways identified to be significantly regulated.** The figure shows signaling pathways and general regulation patterns observed in these, as identified by analysis of each chemical reactivity group. Chemical reactivity abbreviations: Add/El: Addition/elimination to carboxylic acid derivatives; Koor: Coordination bonds; MA: Michael addition or 1,4-Addition to a/b unsaturated carbonyl; NAR: nucleophilic aromatic substitution, PreH: Prehapten; Chemical activation (outside the skin); ProH: Prohapten; Enzymatic activation; SB: Schiff’s Base reaction; SN2: bimolecular nucleophilic substitution. * most significant genes in t-test of negative samples versus given chemical reactivity group. 1) Only pathways where at least one group gives a contribution of 2 times the significance threshold from IPA**®** were reported. ↑: 80% of transcripts or more up regulated, regulated transcripts ≥5. (↑): 80% of transcripts or more up regulated, regulated transcripts <5. ↓: 80% of transcripts or more down regulated, regulated transcripts ≥5. (↓): 80% of transcripts or more down regulated, regulated transcripts <5. MIX: a mix of up and down regulation, regulated transcripts ≥5. (MIX): a mix of up and down regulation, regulated transcripts <5. NC: No significant change compared to non-sensitizers.

**Figure 5:**
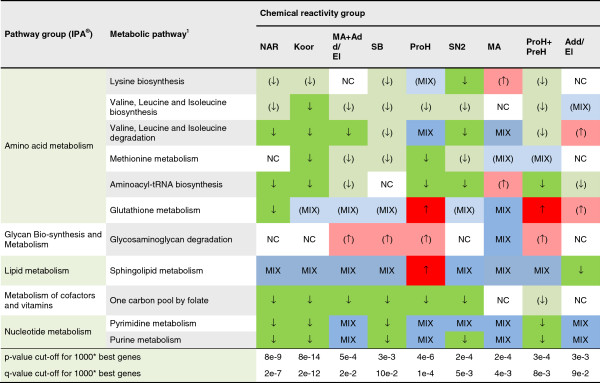
**Metabolic pathways identified to be significantly regulated.** The figure shows metabolic pathways and general regulation patterns observed in these, as identified by analysis of each chemical reactivity group. Chemical reactivity abbreviations: Add/El: Addition/elimination to carboxylic acid derivatives; Koor: Coordination bonds; MA: Michael addition or 1,4-Addition to a/b unsaturated carbonyl; NAR: nucleophilic aromatic substitution, PreH: Prehapten; Chemical activation (outside the skin); ProH: Prohapten; Enzymatic activation; SB: Schiff’s Base reaction; SN2: bimolecular nucleophilic substitution. * most significant genes in t-test of negative samples versus given chemical reactivity group. 1) Only pathways where at least one group gives a contribution of 2 times the significance threshold from IPA**®** were reported. ↑: 80% of transcripts or more up regulated, regulated transcripts ≥5. (↑): 80% of transcripts or more up regulated, regulated transcripts <5. ↓: 80% of transcripts or more down regulated, regulated transcripts ≥5. (↓): 80% of transcripts or more down regulated, regulated transcripts <5. MIX: a mix of up and down regulation, regulated transcripts ≥5. (MIX): a mix of up and down regulation, regulated transcripts <5. NC: No significant change compared to non-sensitizers.

Using the Core Analysis of the pathway software IPA**®**, a number of regulated pathways were identified. In total, 387 regulated pathways were detected in the 9 different chemical reactivity groups. By applying a cut-off of three times, the default IPA**®** significance (−log(p-value) ≤ 3.9), 33 pathways were selected for a comprehensive investigation. The differential engagement patterns of the 33 pathways, related to sensitizer reactivity, are summarized in Figure 3, and detailed in Additional file 1. In addition, Figures 4 and 5 indicate the direction of regulation, as well as the level of significance at which the IPA**®** input gene lists were identified. When studying the 33 selected pathways, it is evident that increasing potency correlates with enhanced pathway involvement in terms of number of regulated genes, as well as a shift towards down regulation of the pathways components.

**Figure 6:**
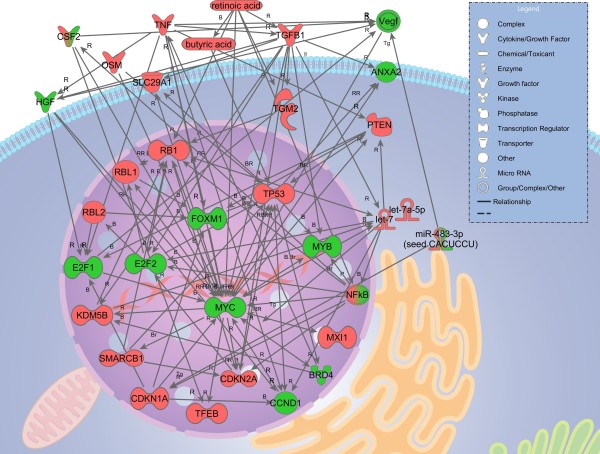
**Network of interactions between transcription regulators observed or predicted in the IPA® analysis of the 9 chemical reactivity groups.** Observed expression patterns were analyzed using IPA® in order to predict upstream regulation. The prediction was based on the expression patterns of the chemical groups and only the most significant predictions were included here. The network shows the known interactions between the regulatory elements identified to be up or down regulated in the analysis. Red color indicates a observed/predicted activation and green color indicates a observed/predicted inhibition of the molecule or complex of molecules. All interactions are based on experimental evidence from the scientific literature. Arrow directions show which way the regulation is directed, if known. R: regulation; B: binding; Br: binding regulated by target; RR: bidirectional regulation; Tg: target. The network was extracted from the MetaCore™ database (Thomson Reuters). For details about observation/prediction state, see Additional files 2, 3, 4 and 5.

Finally, to identify the drivers in relation to potency, analysis of upstream regulators was performed. In Figure 3, the common regulatory elements for all nine groups are summarized. The significance calculations include a predicted activation or inhibition status, which takes regulation of connected molecules into account. Only upstream regulators predicted to be significantly up or down regulated in four or more chemical groups were reported here. The regulatory elements were studied for their relationship to each other, using the MetaCore™ database. These findings were summarized in Figure 6. A complete list of all regulatory elements predicted is found in the Supplementary materials (Additional file 2).

### Prominent pathways and biological functions

The overall impression is that more genes, associated with various pathways, are down-regulated than up-regulated in MUTZ3 cells stimulated with sensitizers. The sensitizers act mainly on cell cycling, cellular stress and injury, and metabolic pathways, in that order. Relevant identified sub functions are apoptosis, cellular growth and development, cellular immune response, intracellular and second messenger signaling, and metabolism and biosynthesis of glycan, lipids, vitamins and cofactors and nucleotides. The general trend is that both metabolic and cell cycle associated pathways are engaged gradually and in correlation with potency. Oxidative stress related pathways are another group of highly engaged pathways. However, these do not correlate with potency in the same manner as the other pathways do. For example, the NRF2-mediated oxidative stress response pathway shows a reversed pattern compared to the other pathways presented in this study. The overall impression of the NRF2 pathway is that phase I and II enzymes, as well as antioxidant proteins, are up-regulated, for an example see Additional file 3. While these trends are quite weak we still think it deserves further investigation. Pathways regulating metabolism and biosynthesis processes are regulated to a lesser extent but still show the same pattern as seen in the cell cycling pathways. Finally, we report 33 potential key regulators of the skin sensitization process found in the analyses. For a complete list of pathways and regulation patterns see Figures 3, 4, and 5. One more pathway has a pattern similar to the one observed in NRF2-mediated oxidative stress response pathway, namely Sphingolipid metabolism. The less potent chemical reactivity groups seem to engage this pathway to a larger extent than the more potent ones.

A few metabolic pathways are generally regulated. These are the nucleotide and lipid metabolism pathways. Chemicals belonging to the nucleophilic aromatic substitution (NAR) and coordination bonds (Koor) group down regulate nucleotide metabolism the most. As the current chemical selection includes only highly potent sensitizers from the Koor and NAR group, we cannot extrapolate these findings to less potent ones. Also, the NAR group constitutes a somewhat odd case, since this is a small group of highly experimental allergens with limited clinical relevance. The other metabolic pathways are regulated in a more mixed fashion, but again, amino acid metabolism is gradually down-regulated with increasing chemical potency. The Glutathione metabolism pathway is interesting in that it goes from clear up regulation by the less potent chemicals to clear down regulation by the NAR group. While the division of pathways into signaling or metabolic pathways is a somewhat arbitrary matter, we still believe it reflects the major cellular functions quite well. We have followed the KEGG standard [19] at the time, adopted from the IPA**®** software.

The pathways of cell cycle regulation are also gradually down-regulated with increasing potency. This could indicate that these chemicals induce cell cycle arrest. As discussed later this seems to be connected to activation state of the up-stream regulators identified in the analyses, especially the *let-7* microRNAs.

The apoptosis pathway Aryl hydrocarbon receptor (AHR) signaling is also among the frequently regulated. As seen from Figure 3, its usage does increase with potency, but the base level is among the highest observed. Several caspases in the AHR signaling are highly up-regulated by many of the chemical groups. Caspases are both involved in cell death processes, like cell shrinkage, membrane blebbing, DNA fragmentation and DNA repair, and in processes of post-translational modifications, protein degradation and synthesis [20]. There are also connections between apoptosis and cell cycle regulators. Caspase 6, for example, is known to interact directly with the transcription factor Rb [21], which is a key regulator of the Cell Cycle Control of Chromosomal Replication pathway. As described previously, a shift towards down regulation of pathway members is observed with increasing potency. For a detailed list of the molecules in the AHR signaling pathway, see Additional file 4.

Finally, lipid metabolism is generally affected by all the chemical groups. For instance, addition/elimination to carboxylic acid derivatives (Add/El) clearly down regulates Sphingolipid metabolism and the remaining chemical groups show a mixed or up-regulated pattern.

### Biological activity of specific chemical groups

From the pathway analysis it is clear that various chemical reactivity groups stimulate pathway activation or inhibition differentially. This selective effect is e.g. observed for chemicals of the Michael addition or 1,4-Addition to a/b unsaturated carbonyl (MA) group, which up regulate several of the signaling pathways, as well as chemicals of the addition/elimination to carboxylic acid derivatives (Add/El). The groups prehapten (Chemical activation outside the skin; PreH) + prohapten (enzymatic activation; ProH) and Add/El (addition/elimination to carboxylic acid derivatives) both show a very limited activity in most pathways studied.

Considering the activity levels and changes of transcription observed and summarized in Figures 3, 4, and 5, we conclude that there is no single mechanism of action of skin sensitizing chemicals. On the contrary, a range of functions are triggered at different levels by the studied chemical groups. Some chemical groups, notably the most potent ones, down regulate most or all pathways studied. Others, like MA, up regulate relatively many pathways, while e.g. addition/elimination to carboxylic acid derivatives (Add/El) and prohapten + prehapten(ProH + PreH) show a quite limited activity except for a few pathways that seem to always be highly regulated. The method also shows promising results in the detection of pre- and prohapten activation as a grouped class of chemical reactivity. However, it does not distinguish between these two activation routes. In the literature cinnamic alcohol is classified as a prohapten [22,23], but recently it has been shown that cinnamic alcohol autoxidize very rapidly [24], making the older classifications uncertain. Therefore, further investigation into the use of methods for separation of these two classes of chemical reactivity is needed. Especially for prohaptens, it is also crucial to confirm purity levels stated by suppliers as even small amounts of impurities may change the reactivity patterns.

It should be noted that all samples in this study are collected at single concentrations and single time-points. Most likely, the observed differences between chemical groups depend in some instances on a varying reactivity speed. As an example, investigations of transcription factors, connecting directly to selected cell cycle pathways, show that the activity level of the pathway molecules can depend on temporal factors. Those chemicals that did not show a great deal of activity in the pathway did indeed have a relatively higher activity level among the upstream regulators of these pathways. Thus, there may be pathways that are more consistently regulated than is observed in this study, although such similarities may be masked by varying reactivity of the chemical sensitizers.

### Upstream regulators

Next, the significantly predicted upstream regulators were investigated. Figure 6 shows the known and experimentally verified interactions in the dataset. The network was manually built, in the IPA**®** software using molecules predicted to be up or down regulated based on analysis of the expression patterns of the nine chemical reactivity groups studied. The molecular interactions were studied in detail using the MetaCore™ software for pathways analyses focusing on uni- or bidirectional binding and regulation actions of the included molecules. All interactions shown in Figure 6 have been experimentally verified elsewhere and are described in the scientific literature. Several of the transcription regulators included in the network bind to each other, e.g. MYC and ANXA2, RB1 and E2f2 and NFkB and E2f2. Central to the network of Figure 6 is a microRNA group named *let-7* (*lethal-7*). The de-regulation of *let-7* miRNAs has previously been described in association to cancer, where the *let-7* family members act as tumor suppressors acting e.g. through MYC and E2f2 [25]. Both MYC and E2f2 were predicted to be down regulated by a majority of the chemical reactivity groups in this study. The microRNA group *let-7* and specifically the member *let-7a-5p* was predicted to be significantly activated in six out of the nine groups, with very high activation scores (z-score 2.6 to 6.6) and regulated with borderline detection in the three remaining groups (z-score 1.3 to 1.8 in ProH, ProH + PreH and Add/El). This finding fits well with the known down regulation of *let-7* by MYC, through binding of MYC to the transcription start site of the *let-7 g pri-miRNA*[26]. *let-7* is known to be active in cell cycle regulation, DNA replication, cell division, apoptosis and development [27]. In the current study, cell cycle, apoptosis, and nucleotide metabolism pathways were found to be down-regulated. This situation is comparable to many forms of cancer, in which low levels of *let-7* have been observed, giving rise to loss of cell cycle control. In our case, down regulation of cell cycle functions is evident for the majority of the chemical reactivity groups studied, while *let-7* was predicted to be up regulated. In a review by Hou et al. [28], *let-7* RNAs were described as heavily down regulated by environmental chemicals, cigarette smoke and up-regulated by others, such as hexahydro-1,3,5-triazine (RDX). *let-7* up-regulation has also been demonstrated in skin samples from psoriasis patients compared to healthy controls [29], suggesting these microRNAs to be key players in skin pathogenesis. Two other, specific, microRNAs were also predicted to be significantly regulated namely *let-7a-5p* and *mir-483-3p*. We propose that these microRNAs may have similar functions, as described above, in skin, when chemically challenged. For details about molecular interactions found see Additional file 5.

*let-7* is known to inhibit a number of molecules: ABCC10, BACH1, BLIMP1 (PRDI-BF1), Bax, CCR7, Dicer, EZH2, HMGA2, HMGI/Y, HOXA9, IL-6, NAV1.9, PGHD, SOCS1, UHRF2, and MYC that are all found regulated in the data set. BCL2L1 and DICER1 were found to be up-regulated while all other were down-regulated. Among the transcription regulators MYC and *let-7* miRNAs are known to bind and interact bi-directionally. Furthermore, *let-7* is regulated by a number of other molecules, several of which were seen regulated in the present analysis, namely PPARA, DROSHA, XPO5, and KDM5B. Finally, *let-7* binds to the predicted activated transcription regulator TGFB1.

The ascribed biological functions of the upstream regulators were extracted from the IPA**®** database. Many of them have known relevant functions in skin and skin disorders, including CDKN1A, CDKN2A, CCND1, CSF2, MYC, PTEN, SMARCB1, TGM2, TP53, E2F2, Retinoic acid, NFkB, TGFB1, TNF-α and *let-7.* Of particular interest, CSF2, NFkB, TGFB1, TNF-α and TP53 have been associated with hypersensitivity and allergy reactions. BRD4, CCND1, CDKN2A, E2F1, FOXM1, HGF, KDM5B. MYB, MYC, NFkB2, PTEN, RB1, RBL1/2, SMARCB1, TGFB1, TP53, Butyric acid and *let-7* are all regulators of the cell cycle. In addition, a majority of these molecules are associated with various forms of cancer, as many carcinomas act through disruption of the cell cycle. Of interest, many of the predicted elements, such as CDKN2A, E2F1, E2F2, MYC, SMARCB1, TP53, TGM2, TNF-α, Retinoic acid, NFkB and TGFB1 are known regulators or members of the Aryl Hydrocarbon Receptor signaling pathway.

The NFkB complex was also predicted to be activated in this study. This extensively studied nucleic protein has a wide range of functions. Of interest to this study is mainly its involvement in pathways of Xenobiotics Metabolism, Production of Nitric Oxide and Reactive Oxygen Species in Macrophages and Toll like receptor signaling. NFkB showed a heterogeneous pattern, with up regulation in four groups (MA + Add/El, MA, SN2 and ProH) and down regulation in one (ProH + PreH). This may be a relevant finding given the role this molecule plays in epithelial cells where it on one hand maintains immunological homeostasis and on the other induces inflammatory processes [30]. NFkB has been shown to be able both to activate and inhibit inflammatory pathways in skin [31], a finding that might explain the mixed pattern seen in this study.

### Implications of the results

Activation of MUTZ-3 cells with skin sensitizers resulted in regulation of a number of previously known, as well as novel, canonical pathways. The 33 most significantly regulated pathways were studied in detail. After stratification of sensitizing chemicals into chemical reactivity groups, it was evident that pathway engagement and the number of regulated molecules within such pathways were linked to the sensitizing potency of the stimulatory agent. For instance, cell cycle associated pathways were engaged gradually in correlation with the sensitizing potency of the chemicals used for cell stimulations. These observations can be utilized when designing tools for assessment of sensitizing potency of chemicals in predictive assays. Currently, the primary prediction call obtained from GARD is binary, i.e. compounds are classified as sensitizers or non-sensitizers. However, this readout can now for the first time be complemented with a potency call based on mechanistic information obtained through pathway analysis. While implementation of such classifications are not straight-forward, similar attempts have been done within cancer research, where pathway activity has successfully been associated with subtypes of cancer and progression of disease [32-34].

Oxidative stress related pathways are also in the group of highly engaged pathways. These do not correlate with potency to the same extent that cell cycle pathways do. For example, the NRF2-mediated oxidative stress response pathway shows a higher engagement by less potent chemical groups compared to more potent ones. This may be a consideration when studying more clinically relevant allergens, as these are rarely in the strong to extreme but rather in the week to medium range of potency.

Pathways regulating metabolism and biosynthesis processes constitute the third large group of canonical pathways identified to be regulated in skin sensitization. These pathways are regulated to a lesser extent but still show the same pattern as seen in the cell cycling pathways.

No canonical pathway was unanimously regulated within each data set. The importance of this fact becomes apparent when considering the design of various proposed tests for animal-free assessment of sensitization, which today are mainly based on analysis of single biomarkers. It is evident that excessively simplistic tests lack the power to accurately assess compounds as sensitizers or non-sensitizers, if the target chemical domain is wide. Recognition of the specific subtype of chemicals to be tested based on reactive mechanism or otherwise, will be crucial in order to train and configure predictive models accurately.

## Conclusions

In the present transcriptional profiling study, we have shown that skin sensitizers trigger a large number of signaling and metabolic pathways in MUTZ-3 cells. Among these, we found examples of well-known pathways, as well as pathways previously not described in relation to chemical sensitization. Importantly, a majority of the pathways described show a clear correlation with both chemical reactivity and sensitizing potency.

The strongest correlation between pathway usage and potency is found in the cell cycle, nucleotide metabolism and cancer pathways. As these groupings are obviously rather arbitrary it is probably more correct to say that functions involving cell cycle regulation and cell cycle control both in healthy and diseased conditions correlate very well with chemical potency. Nucleotide metabolism is tightly connected to the cell cycle and consequently a down regulation of nucleotide metabolism in parallel to a down regulation of cell cycle functions is expected. The apoptosis pathway Aryl Hydrocarbon Receptor signaling is a novel finding with regard to potency prediction. This pathway is among the highly regulated ones but still shows a decrease in activity with decrease in potency.

Finally, transcription regulation of the sensitization process was investigated, by prediction of activation and inhibition of upstream regulators. A network of regulatory elements that seemed to be generally involved was identified. These seemed to be centered on the transcription regulator MYC and the miRNA *let-7*.

The presented results have implications for the design of predictive *in vitro* test for assessment of sensitization potency. Few chemicals appear to induce identical responses *in vitro*, indicating that excessively simplistic assays will fail to correctly classify unknown samples. Subdivisions among sensitizers, by reactive domain or otherwise, should be considered with caution and predictive models should be trained accordingly. In brief, we demonstrate for the first time that genomic approaches contain valuable mechanistic information associated with potency assessment of chemicals, which is in line with the current demand of evidence based toxicology.

## Abbreviations

ACD: Allergic contact dermatitis; Add/El: Addition/elimination to carboxylic acid derivatives; AHR: Aryl Hydrocarbon Receptor; HCA: Hierarchical cluster analysis; Koor: Coordination bonds; LLNA: Local Lymph Node Assay; MA: Michael addition or 1,4-Addition to a/b unsaturated carbonyl; NAR: Nucleophilic aromatic substitution; PCA: Principal component analysis; PreH Prehapten: Chemical activation (outside the skin); ProH Prohapten: Enzymatic activation; SB: Schiff’s Base reaction; SN2: Bimolecular nucleophilic substitution.

## Competing interests

The authors declare no conflicts of interests.

## Authors’ contributions

ML and CB designed the sensitization study strategy. HJ and ML set up and optimized the cell-based assay. HJ performed the cellular stimulations with chemicals. AA, HJ and ML designed the analysis strategy. AA performed the data analysis and prepared the text and figures. AB contributed with chemical knowledge and data set stratification guidelines. AA, HJ and ML drafted the manuscript. All authors revised and approved the manuscript. All authors read and approved the final manuscript.

## Pre-publication history

The pre-publication history for this paper can be accessed here:

http://www.biomedcentral.com/2050-6511/15/5/prepub

## Supplementary Material

Additional file 1 **Table of gene involvement shown per reactivity group and per pathway, used for Figure **3. For each pathway described in Figure 3 the exact numbers of molecules involved are given in this table. No distinction is made between up or down regulated molecules. Click here for file

Additional file 2 **Table of upstream regulators predicted to be up or down regulated in the IPA® analysis.** Predictions were based on expression data from the nine chemical reactivity groups studied. Click here for file

Additional file 3 **NRF2 Oxidative Stress Pathway map for Michael addition or 1,4-Addition to a/b unsaturated carbonyl reactive chemicals.** The pathway map shows the NRF2 Oxidative Stress Pathway with expression data from the 1000 most significant genes regulated by MA. Red shows up regulation and green down regulation of a given molecule. The less significantly regulated molecules were not shown in this figure, indicated here as white molecule symbols. Click here for file

Additional file 4 **A detailed list of molecules in the Aryl Hydrocarbon Receptor (AHR) signaling pathway.** The file shows all the molecules included in the AHR signaling pathway, as defined by IPA®. Common identifiers are included to enable analysis in any software. Click here for file

Additional file 5 **All upstream regulators identified in pathway analysis in IPA®.** This file contains the complete result from the prediction analysis of the expression patterns seen in the nine data sets, one from each chemical reactivity group studied. The upstream regulators were predicted from the pattern of up or down regulated target molecules. Significance of finding is given as z-scores for each upstream regulator and an attached predicted activation status shows if the molecule is predicted to be up or down regulated. Molecules measured on the microarrays (observed) are recognized by a value in the fold change column. Click here for file
