# Supplementary material for: Skin sensitizers differentially regulate signaling pathways in MUTZ-3 cells in relation to their individual potency
Source: BMC Pharmacol Toxicol. 2014 Feb 11;15:5. doi: 10.1186/2050-6511-15-5 (PMC3932014; doi:10.1186/2050-6511-15-5)
Supplement: Additional file 2 — Table of upstream regulators predicted to be up or down regulated in the IPA® analysis. Predictions were based on expression data from the nine chemical reactivity groups studied. [file 2050-6511-15-5-S2.docx]

**Table 1**. The predicted upstream regulating factors from the analysis of MUTZ-3 cells stimulated by chemicals of nine different reactivity types.

| Upstream Regulator | Observed/Predicted Activation State | Regulated by # of groups (above threshold) | Synonym | Regulator type | Chemical reactivity groups regulated |
| --- | --- | --- | --- | --- | --- |
| ANXA2* | Inhibited | 5 | CAL1H | Ca-dependent phospholipid-binding protein | Koor  MA  MA+Add-El  ProH  SB  NAR  SN2 |
| Butanoic acid | Activated | 6 | Butyrate, CAS no. 107-92-6 | endogenous chemical | Koor  MA  MA+Add-El  ProH  SB  SN2  NAR  ProH+PreH |
| BRD4 | Inhibited | 7 | HUNK1 | kinase | Add-El  Koor  NAR  ProH  ProH+PreH  SB  SN2  MA+Add-El |
| CCND1* | Inhibited | 4 | Cyclin D1 | cyclin | Koor  MA+Add-El  NAR  SB  SN2 |
| CDKN1A* | Activated | 4 | P21 | transcription regulator | MA+Add-El  NAR  SB  SN2  Koor  ProH |
| CDKN2A* | Activated | 6 | p14ARF (isoform4), p16INK4 (isoform 1, 2, 3) | transcription regulator | Koor  MA+Add-El  NAR  ProH+PreH  SB  SN2  ProH |
| CSF2 | Mixed, Activated in genes above threshold | 4 |  | cytokine | MA  MA+Add-El  ProH  SN2  Add-El  NAR |
| E2F1 | Inhibited | 4 | RBAP1 | transcription regulator | Koor  MA+Add-El  NAR  SB  MA  ProH  ProH+PreH  SN2 |
| E2F2* | Inhibited | 5 | - | transcription regulator | Koor  MA+Add-El  NAR  ProH  SB  MA  SN2 |
| FOXM1* | Inhibited | 4 | FOCM1, forkhead box M1 | transcription regulator | MA+Add-El  NAR  SB  SN2  ProH |
| HGF | inhibited | 4 |  | growth factor | Add-El  MA+Add-El  NAR  SB  ProH  ProH+PreH  SN2 |
| KDM5B* | Activated | 5 | PLU-1 | transcription regulator | Koor  MA+Add-El  NAR  ProH  SB  SN2 |
| let-7 | Activated | 6 | - | microRNA | Koor  MA  MA+Add-El  NAR  SB  SN2  Add-El  ProH  ProH+PreH |
| let-7a-5p (and other miRNAs w/seed GAGGUAG) | Activated | 5 | MIRLET7A | mature microRNA | Koor  NAR  SB  SN2  Add-El  MA  MA+Add-El  ProH  ProH+PreH |
| miR-483-3p (miRNAs w/seed CACUCCU) | Mixed | 4 | MIR483 | microRNA | MA (inhibited)  MA+Add-El  NAR  SB |
| MXI1 | Activated | 4 | MAD2, Max interactor 1 | transcription regulator | Koor  MA+Add-El  NAR  ProH  MA  SB  SN2 |
| MYB* | Inhibited | 4 | c-Myb, v-Myb | transcription regulator | Koor  MA+Add-El  NAR  SB  ProH  SN2 |
| MYC* | Inhibited | 8 | c-Myc, v-Myc | transcription regulator | Koor  MA  MA+Add-El  NAR  ProH  ProH+PreH  SB  SN2 |
| NFkB (complex) | Mixed | 4 | - | Complex including NFKB1, NFKB2, REL, RELA, RELB | MA (inhibited)  MA+Add-El  SN2  ProH |
| OSM | Activated | 5 | OncoM | cytokine | MA  MA+Add-El  NAR  ProH  SN2 |
| PTEN | Activated | 5 | GLM2 | phosphatase | MA+Add-El  NAR  ProH  SB  SN2  Koor  MA |
| RB1 | Activated | 5 | OSRC | transcription regulator | MA+Add-El  NAR  ProH+PreH  SB  SN2  Koor  ProH |
| RBL1* | Activated | 6 | P107 |  | Koor  MA+Add-El  NAR  ProH  SB  SN2  MA  ProH+PreH |
| RBL2 | Activated | 4 | P130 |  | MA+Add-El  NAR  ProH  SB  Koor  MA  SN2 |
| Retinoic acid | Activated | 8 | tretinoin, CAS: 302-79-4 | endogenous chemical | Koor  MA  MA+Add-El  NAR  ProH  ProH+PreH  SB  SN2 |
| SLC29A1* | Activated | 5 | ENT1 | transporter | Koor  MA  MA+Add-El  ProH  SB |
| SMARCB1 | Activated | 5 | BAF47 | transcription regulator | MA  MA+Add-El  NAR  ProH  SB  SN2 |
| TFEB | Activated | 4 | - | transcription regulator | MA  ProH  ProH+PreH  SN2  MA+Add-El |
| TGFB1 | Activated | 6 | TGFB | growth factor | MA  MA+Add-El  NAR  ProH  SB  SN2  ProH+PreH |
| TGM2 | Activated | 7 | TGASE | enzyme | Koor  MA  MA+Add-El  NAR  ProH  SB  SN2  ProH+PreH |
| TNF | Activated | 4 |  | cytokine | MA  MA+Add-El  ProH  SN2  ProH+PreH |
| TP53 | Activated | 8 | P53 | transcription regulator | Koor  MA  MA+Add-El  NAR  ProH  ProH+PreH  SB  SN2 |
| Vegf | Inhibited | 5 | - | Group including FIGF, PDGFC, PGF, PROK1, VEGFA, VEGFB, VEGFC | Add-El  Koor  MA+Add-El  NAR  SB  MA  ProH  ProH+PreH  SN2 |

The table shows the upstream regulators predicted to be activated in the data set. Only molecules predicted to be activated or inhibited in five or more chemical groups are shown. A complete list of upstream regulators for each chemical group can be found in Additional file 4.

*Indicates shows that this molecule was measured on the array, here denoted as observed, in contrast to the predicted molecules, calculated to be present by the IPA program. For details about observation/prediction state, see Additional file 5. Observation state was not show for complexes of molecules.
