# Supplementary material for: Skin sensitizers differentially regulate signaling pathways in MUTZ-3 cells in relation to their individual potency
Source: BMC Pharmacol Toxicol. 2014 Feb 11;15:5. doi: 10.1186/2050-6511-15-5 (PMC3932014; doi:10.1186/2050-6511-15-5)
Supplement: Additional file 3 — NRF2 Oxidative Stress Pathway map for Michael addition or 1,4-Addition to a/b unsaturated carbonyl reactive chemicals. The pathway map shows the NRF2 Oxidative Stress Pathway with expression data from the 1000 most significant genes regulated by MA. Red shows up regulation and green down regulation of a given molecule. The less significantly regulated molecules were not shown in this figure, indicated here as white molecule symbols. [file 2050-6511-15-5-S3.docx]

**
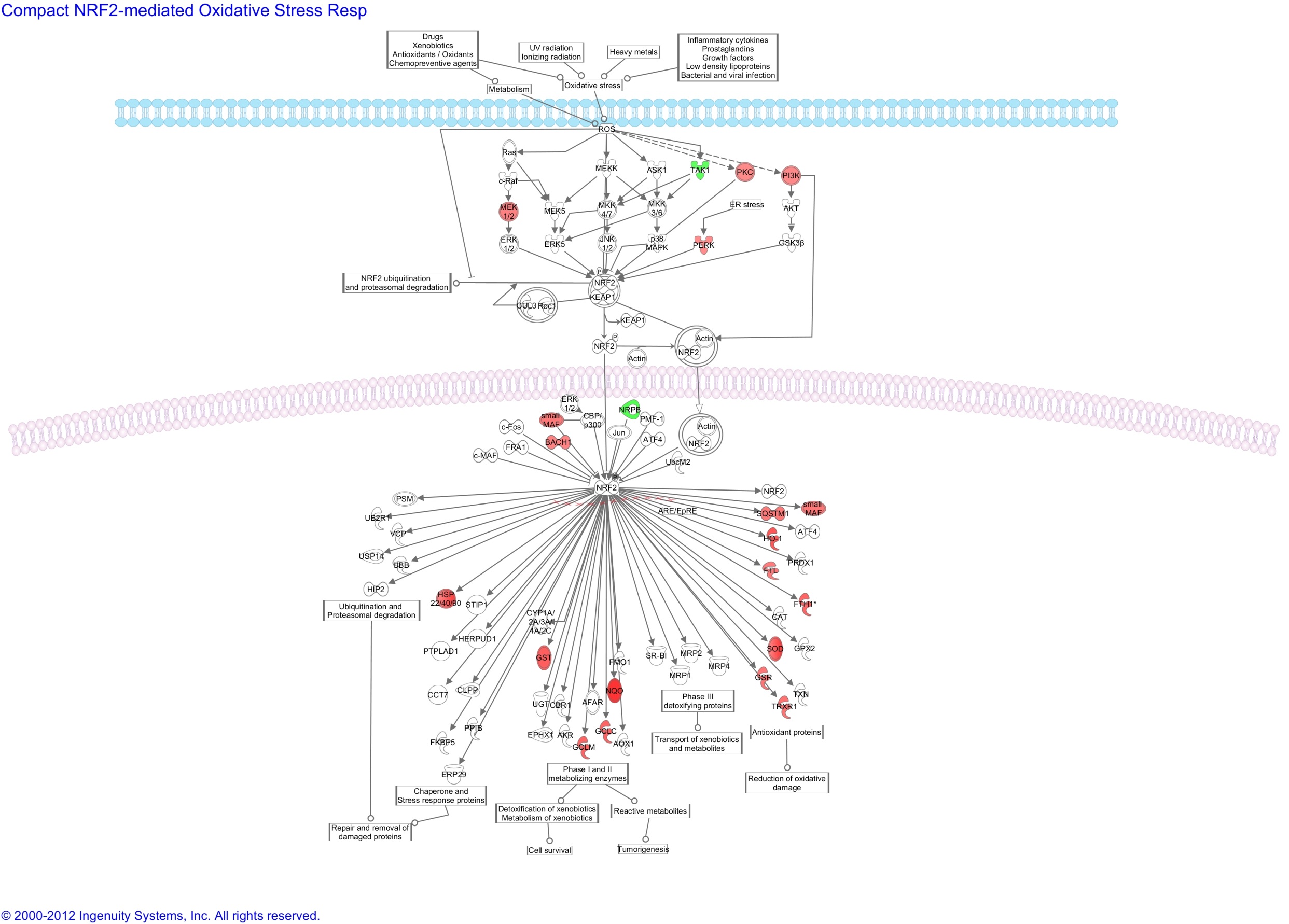
**

**Figure 2**

The NRF2 Oxidative Stress Response pathway with expression data for the MA chemical group. Data from the 1000 most significant genes is shown here with green showing down regulation and red up regulation. The strongest signal in the MA reactivity group is the up regulation of phase I and II enzymes and antioxidant proteins. The same pattern is induced by the ProH and ProH+PreH chemical groups while other chemical groups show a somewhat response pattern where the dominating pattern is a down regulation of the chaperone proteins (e.g. SN2 and SB). The key regulator of the pathway, NRF2, is found up regulated among the top 2000 genes and is therefore not colored in this graph.
